# Epitome of Sanitary Literature

**Published:** 1858-01

**Authors:** 


### THE EPITOME OF SANITARY LITERATURE.

The past quarter, although the great publishing quarter, has brought forth but few books bearing specially on sanitary science; and from such as have been published, we have but space to select two or three for analytical remark.

Dr. Aitken * has brought out an admirable *Hand-book of Medicine,* which we strongly recommend to our medical readers, and to such general scientific readers as are interested in the advancement of the curative art. There is a novelty in this work, in the fact that the author has accepted Dr. Farr's classification of diseases, as adopted by the Registrar General. We have already expressed an opinon in reference to this nosological system. In the last part of the work there is an excellent description of the geographical distribution of disease, after the plan of Mr. Keith Johnstone.

M. Baudens has published a work on the *Sanitation of the French Army in the Crimea,* which is full of valuable details. It must be reserved for a future occasion.

Dr. Cockle,† in an eloquent and thoughtful address on *General Pathology,* delivered before the students of the Grosvenor Place School of Medicine, indicates the relationships of pathology to the geographical distribution of disease. The following quotation is in point:—

"Pathology creates, as it were, the necessity of a Medical Geography, by its inquiries respecting the direct influence of soil and climate upon the duration of life, as well in accelerating as in retarding the progress of given diseases. Indeed, this latter science bids fair to take a position of the highest order, and has already determined some remarkable results. I shall cite one or two of such from the recently published work of Boudin. He says there are some types of races which appear to adapt themselves to climatic change in a wonderful manner, while others again scarcely endure the slightest alteration of habitat. The Jews may be cited as examples of the first order. They are, at present, spread over all parts of the world. In Europe they are found from Gibraltar to Norway; in Africa, from Algiers to the Cape of Good Hope; in Asia, from Cochin to the Caucasus, and from Java to Pekin; in America they are met with from Montevideo to Quebec. For the last half century they have become residents of Australia, and have given proof of being thoroughly acclimatised under the tropics,

* Handbook of Medicine. By W. Aitken, M.D. London: Griffin. 1857.
† Introductory Lecture to a Course of General Pathology. By John Cockle, M.D. Pamphlet. London: T. Richards. 1857.

where a population of European origin have constantly failed to perpetuate themselves. The Jew has also lived through a long series of ages, and still lives, upon the only point of the globe, the valley of Jordan, situated more than 4000 *mètres* below the level of the sea; a spot where it becomes matter of doubt whether the European could ever propagate his race. Finally, wherever the Jewish race has been studied to the present time, the statistical laws of births and deaths, proportion of sexes, etc., which affect it, have been proved to be completely different from those which preside over other nationalities in the midst of which it resides.

"In opposition to this cosmopolitanism of the Jewish race, look, on the other hand, to the continuous decrease of an European population in all tropical countries, and to the impossibility, hitherto ascertained, of perpetuating itself in Egypt and several other parts of Africa. In the tables published by the French Minister of War, the mortality of the population, which in France is 24 in 1000, and which, in 1849, notwithstanding the cholera, did not even attain the proportion of 28 in 1000, has increased in Algeria in the following proportion:—

| | Deaths in 1000. |
|---|---|
| 1848 .. .. .. .. | 41 |
| 1849 .. .. .. .. | 101 |
| 1850 .. .. .. .. | 70 |
| 1851 .. .. .. .. | 64 |
| 1852 .. .. .. .. | 55 |
| 1853 .. .. .. .. | 47 |

And so late as 1854, the last year that reports have been published, the deaths among European population amounted to 7025, while the births were only 6111.

"Still to linger in this interesting field one moment longer; how curious is the law of antagonism that appears to exist between ague and consumption. Dr. Green, of Whitehall, in the province of Washington, United States, where intermittents are of unusual frequency, declares that there did not exist one single instance of phthisis developed in the district; and that the consumptive invalids arriving there experienced a relief as decided as it was permanent. The same author also states that a morass near Rutland, having been converted into a pond, the intermittent fevers disappeared from that part of the country, and were replaced by pulmonary consumption. The population having solicited and obtained the suppression of the pool, or, what amounted to the same thing, the re-establishment of the morass, the original agues returned and antagonised the development of phthisis. These facts are supported by the experience of many other competent observers. I need not say that they demand your serious consideration, especially at a period when human migration is going on on so large a scale." (pp. 11-12.)

